# Blood and Tissue Advanced Glycation End Products as Determinants of Cardiometabolic Disorders Focusing on Human Studies

**DOI:** 10.3390/nu15082002

**Published:** 2023-04-21

**Authors:** Yoona Kim

**Affiliations:** Department of Food and Nutrition, Institute of Agriculture and Life Science, Gyeongsang National University, 501 Jinju-daero, Jinju 52828, Gyeongsangnam-do, Republic of Korea; yoona.kim@gnu.ac.kr; Tel.: +82-55-772-1432

**Keywords:** dietary advanced glycation end products, cardiometabolic disorders, skin auto fluorescence

## Abstract

Cardiometabolic disorders are characterised by a cluster of interactive risk determinants such as increases in blood glucose, lipids and body weight, as well as elevated inflammation and oxidative stress and gut microbiome changes. These disorders are associated with onset of type 2 diabetes mellitus (T2DM) and cardiovascular disease (CVD). T2DM is strongly associated with CVD. Dietary advanced glycation end products (dAGEs) attributable from modern diets high in sugar and/or fat, highly processed foods and high heat-treated foods can contribute to metabolic etiologies of cardiometabolic disorders. This mini review aims to determine whether blood dAGEs levels and tissue dAGEs levels are determinants of the prevalence of cardiometabolic disorders through recent human studies. ELISA (enzyme-linked immunosorbent assay), high-performance liquid chromatography (HPLC), liquid chromatography-mass spectrometry (LC-MS) and gas chromatography-mass spectrometry (GC-MS) for blood dAGEs measurement and skin auto fluorescence (SAF) for skin AGEs measurement can be used. Recent human studies support that a diet high in AGEs can negatively influence glucose control, body weight, blood lipid levels and vascular health through the elevated oxidative stress, inflammation, blood pressure and endothelial dysfunction compared with a diet low in AGEs. Limited human studies suggested a diet high in AGEs could negatively alter gut microbiota. SAF could be considered as one of the predictors affecting risks for cardiometabolic disorders. More intervention studies are needed to determine how dAGEs are associated with the prevalence of cardiometabolic disorders through gut microbiota changes. Further human studies are conducted to find the association between CVD events, CVD mortality and total mortality through SAF measurement, and a consensus on whether tissue dAGEs act as a predictor of CVD is required.

## 1. Introduction

Human studies showed the association between plasma concentrations of advanced glycation end products (AGEs) and cardiometabolic disorders [1,2,3]. The association between higher blood circulating AGEs levels and higher insulin resistance was observed [2,3], while other studies [4,5,6] showed the association between lower blood circulating AGEs levels and higher insulin resistance. Moreover, AGEs accumulation in tissues can increase risk of cardiovascular disease (CVD), especially in subjects with diabetes mellitus and coronary artery disease [7].

Several papers [8,9] have reviewed the purpose of dietary advanced glycation end products (dAGEs) on cardiometabolic disorders. Clarke et al. 2016 [8] conducted a systematic review of 12 randomised controlled trials (11 publications [10,11,12,13,14,15,16,17,18,19,20]) to examine dAGEs on risk for chronic disease. They concluded that AGEs high in diet appeared to increase risk for chronic disease attributable from oxidative stress and inflammation. Luévano-Contreras et al. 2017 [9] concluded that dAGEs contributed to risk for cardiometabolic disorders by endogenously interacting with AGEs. A diet high in N-epsilon-(Carboxymethyl) lysine (CML) elevated endothelial dysfunction and arterial aging. Subjects with type 2 diabetes mellitus (T2DM) or higher risk for cardiometabolic disorders who consumed high dAGEs were more likely to have increased oxidative stress and markers of inflammation and endothelial dysfunction [9].

Therefore, the aims of this mini review are as follows: (a) to examine the effect of dietary blood AGEs levels on cardiometabolic disorders based on recent human studies; (b) to examine the role of skin AGEs levels on cardiometabolic disorders.

## 2. Advanced Glycation End Products

### 2.1. AGEs Formation

AGEs are stable, irreversible end products generated from nonenzymatic reaction between reducing sugars with amino groups or lipids or nucleic acids through the Maillard reaction forming Schiff base/Amadori products which can be promoted by high temperature, resulting in features of yellow-brown colour [21,22]. AGEs can be endogenously formed but can be exogenously sourced from diet (highly processed, high temperature-treated foods), pollution and smoking [23,24,25,26]. The high-temperature cooking techniques such as dry heat (e.g., roasting, grilling, baking and deep-frying) can lead to the Maillard reaction [27]. The quantity of AGEs consumed is obviously determined by the type of food, cooking method and degree of processing in industry [24,28]. Foods high in lipid and protein (e.g., particularly cheese, butter and red meat) when treated by dry heat contain a high quantity of AGEs [24,29]. Food items processed in industry (e.g., baked, bakery products) contain considerably high AGEs [30], while whole grains, milk, fruits and vegetables are relatively low in AGEs [24].

The endogenous formation of AGEs is involved in several pathways (non-enzymatic Maillard reaction, polyol pathway and lipid peroxidation) [31,32,33]. AGEs can be endogenously produced as products of Schiff base/Amadori products or/and through the polyol pathway [32]. A number of chemical compounds can be generated through the Maillard reaction. These chemical compounds are classified into reactive intermediates and stable end products of AGEs [32]. The Maillard reaction can occur; the early step includes condensation resulting from non-enzymatic reaction between carbonyl groups of reducing sugars (aldose or ketose) and amine groups of nucleic acids, proteins (free arginine, lysine, cysteine, histidine and tryptophan), or lipids, leading to endogenous formation of reversible intermediate products such as Schiff’s base, and then Amadori rearrangement and subsequent glycoxidation for AGEs production [34]. AGEs are a group of heterogeneous, cross-link formation through glycation process. The AGEs formation can be promoted in persistent hyperglycaemia (e.g., in diabetes mellitus) [35]. Elevated glucose levels are associated with oxidative stress, glucose auto oxidation and activated polyol pathway [36,37]. Hyperglycaemia condition promotes lipid peroxidation leading to production of advanced lipid peroxidation end products (ALEs) [38,39]. In addition, the Namiki pathway (aldimins) and the Wolff pathway (auto-oxidation of carbonyl compounds or monosaccharides) through reactive oxygen species (ROS) or transition metals can be involved in AGEs production [40]. Dicarbonyl compounds (highly reactive molecules of protein cross-links) such as glyoxal, methylglyoxal (MG) and 3-deoxyglucosone can be produced through oxidative degradation or auto-oxidation of Amadori products [41]. Several AGEs are not exclusively produced through the Maillard reaction. Especially, CML (glucose-lysine or threose-lysine) can be generated through oxidative degradation of Amadori products or direct reaction of glyoxal with lysine and lipid peroxidation [42,43].

### 2.2. Characteristics of AGEs

The properties and chemical structures of AGEs (e.g., fluorescent, cross-linking and weights) can be determined through several pathways and/or various AGEs precursors involved in AGEs production. AGEs can be classified with several groups based on their sources, precursors non-fluorescent or fluorescent, non-cross-linked or cross-linked, molecular weight, receptor for AGEs (RAGE) ligands and physiological importance (toxic or non-toxic) [34]. Pentosidine is a fluorescent glycoxidation product and protein–protein cross-linked AGEs. Argpyrimidine is a fluorescent and non-cross-linked AGE. N-epsilon-(1-carboxyethyl) lysine (CEL), CML and pyrraline are non-fluorescent and non-cross-linked AGEs. Glyoxyl-lysine dimer (GOLD), methylglyoxal-lysine dimer (MOLD) and 3-deoxyglucosone-lysine dimer (DOLD) are non-fluorescent and cross-linked AGEs [34].

AGEs less than 12 kDa are classified as low weight molecules. AGEs greater than 12 kDa are regarded as large weight molecules. High-molecular AGEs are known as protein-binding proteins, and small-molecular AGEs are known as free proteins or peptide-binding molecules. Low-molecular-weight AGEs can become high molecules by heat treatment [44,45].

The extent of dAGEs absorption depends on their chemical structures, molecular weights and hydrophobicity (e.g., pyrraline and argpyrimidine). Highly hydrophobic AGEs easily pass through the basolateral membrane compared with hydrophilic AGEs (e.g., CML, CEL and MG-H1) [34,46,47]. Approximately one-third of AGEs are excreted in the urine. However, less than 5% of AGEs are excreted in patients with diabetes [48]. 

Circulating AGEs contents in the whole body can be attributed by the balance between endogenous AGEs synthesis, AGEs tissue accumulation, exogenous AGEs absorption from food and AGEs clearance in the kidney [7,24,49,50]. Plasma AGEs levels have been used as biomarkers to examine the incidence of cardiometabolic disorders. 

Several potential treatment options for AGEs-mediated cardiometabolic disorders are recognised as follows: (a) AGEs formation inhibition [e.g., aminoguanidine, 2, 3-diamino-phenazine, tenilsetam, pyridoxamine, arginine, 2-isopropylidenehydrazono-4-oxo-thiazolidin-5-ylacetanidide (OPB-9195); (b) breaking down Maillard cross-links [e.g., 4, 5-dimethyl-3-phenacylthiozolium chloride (ALT-711), N-(2-acetamidoethyl) hydrazine carboximidamide hydrochloride (ALT-946), N-phenacetylthiazolium bromide (PTB) and alagebrium]; (c) RAGE inhibitors (e.g., anti-RAGE antibodies, statins and curcumin); (d) redox regulators (e.g., aldose reductase inhibitors, angiotensin-converting enzyme inhibitors and angiotensin II receptor blockers); (e) antioxidants (e.g., vitamin C, vitamin E, α-tocopherol, taurine, niacinamide, oxerutin pyridoxal, lipoic acid, N-acetylcysteine and epigallocatechin-3-gallat) [51,52,53,54,55,56,57,58,59].

### 2.3. AGEs Measurements

Various AGEs quantification techniques have been developed, including biochemical and immunohistochemical methods to measure pentosidine and CML. ELISA (enzyme-linked immunosorbent assay) utilising AGEs-recognising antibodies (Ac) have been designed [60,61]. Moreover, high-performance liquid chromatography (HPLC), liquid chromatography-mass spectrometry (LC-MS) and gas chromatography-mass spectrometry (GC-MS) can determine plasma concentrations of AGEs according to the fluorescence intensity [62,63,64]. Skin auto fluorescence (SAF) can assess AGEs in skin [65,66,67].

## 3. Dietary Patterns Influence on Carbonyl Stress or Blood AGEs Levels

Certain diets low in AGEs may attenuate a total AGEs level in the body contributing to a lower risk of cardiometabolic disorders. In observational studies [48,68], a diet high in AGEs (AGEs, CML, CEL and MG-H1) was associated with higher concentrations of CML, CEL and MG-H1 in plasma and urine [48]. Maasen et al.al. 2019 [68] conducted a cross-sectional Diabetes and Atherosclerosis Maastricht (CODAM) study of 574 subjects with a moderately increased risk of T2DM and CVD, in order to examine the effect of carbohydrate quality [glycaemic index (GI)] and quantity [glycaemic load (GL)] in a habitual diet on dicarbonyls and AGEs. They found the association between higher GL diet and higher levels of free urinary MG-H1 [68].

In randomised controlled trials (RCTs) [69,70], Delgado-Andrade et al. 2011 [69] examined the influence of Millard reaction products (MRP) diet on CML (one of the most biologically active MRP), consumption and excretion comparing a MRP-high diet with a MRP-low diet in 20 healthy male adolescents aged 11–14 years. A MRP-high diet (high in processed browning food: chocolates, corn flakes, baked products, fried and breaded foods) elevated CML levels and CML faecal excretion compared with a MRP-low diet, indicating that dietary CML levels affect CML absorption and CML faecal excretion [69]. Brinkley et al. 2020 [70] showed the association between higher dietary protein intake (≥1.2 g/kg/d) and over 5–10% higher levels of CML and soluble RAGE (sRAGE), compared with lower protein intakes in older subjects aged mean 73 years [70].

The Mediterranean diet is a dietary pattern rich in whole grains, legumes, nuts, vegetables, fruits and olive oil including a moderate amount of seafood, fish, dairy and red wine, and less amount of processed meat and red meat. This Mediterranean diet constitutes low in saturated fat, animal protein and high in monounsaturated fatty acids (MUFAs), antioxidants and fibre, and proper balance between omega-6 and omega-3 fatty acid [71,72]. Mediterranean diets [73,74] or Mediterranean-type diets [rich in MUFAs [75] with a low AGEs content have been shown to reduce circulating AGEs, decrease oxidative stress and have anti-inflammatory effects. Gutierrez-Mariscal et al. 2020 [73] observed that a 5-year intake of a Mediterranean diet decreased blood AGEs levels (especially, MG levels), which was associated with improved beta cell functionality index assessed by the disposition index (DI) in newly diagnosed T2DM subjects with coronary heart disease (CHD), compared with intake of a low-fat diet. This indicated that a Mediterranean diet could play a T2DM remission role through enhanced beta cell function attributable to reduced circulating AGEs levels. Lopez-Moreno et al. 2016 [74] conducted a RCT of 20 subjects aged over 65 years in a crossover design. Subjects underwent three 4-week isocaloric diet interventions. A meal test was performed at the end of each 4-week intervention. Three isocaloric diets included the Mediterranean diet, saturated fat diet and omega-3 diet consisting of low in fat and high in carbohydrate. The Mediterranean diet was comprised of 38% fat [24% MUFA from virgin olive oil; 10% saturated fatty acids (SFA); 4% polyunsaturated fatty acids (PUFA)]. The saturated fat diet was comprised of 38% fat (12% MUFA; 22% SFA; 4% PUFA). The omega-3 diet was comprised of 28% fat (10% SFA; 12% MUFA; 8% PUFA with 2% alpha-linolenic acid). The Mediterranean diet reduced levels of sMG, sCML and RAGE mRNA (oxidative stress and inflammation inducer) and increased levels of AGE receptor 1 (AGER1: AGEs clearance with AGEs endocytic uptake and degradation), and GloxI (antioxidant defence marker) in the fasting and postprandial states, compared with the SFA diet or the omega-3 diet [74]. Lopez-Moreno et al. 2017 [75] conducted a RCT in 75 subjects with metabolic syndrome. Subjects with metabolic syndrome were randomly allocated to one of four isoenergetic diets for 12 weeks. Four diets are as follows: (a) a high-fat, saturated fatty acid-rich diet [38% fat (16% SFA)]; (b) a high MUFA diet [38% fat (20% MUFA from olive oil)]; (c) low-fat, high-complex carbohydrate-rich diets with placebo capsule [1.2 g/day of control high-oleic sunflower seed oil capsules (placebo)]; (d) low-fat, high-complex carbohydrate-rich diets with a 1.24 g/day of n-3 capsule (long-chain (n-3) polyunsaturated fatty acid [ratio of 1.4 eicosapentaenoic acid (EPA):1 docosahexaenoic acid (DHA)]. Meal tests representing each intervention diet were performed at the end of each 12-week diet. Reductions in blood levels of AGEs and RAGE mRNA and increases in blood mRNA levels of AGER1and GloxI were observed after a 12-week high MUFA diet, compared with other diets. These findings indicated a low-AGEs diet rich MUFA could alter AGEs metabolism by increasing AGER1 which can decrease AGEs levels in the intracellular and extracellular spaces as an antioxidant [75]. 

## 4. Dietary AGEs Influence on Glucose Homeostasis

AGEs can cause pancreatic β cell failure by suppressing insulin production and secretion, causing abnormal glycaemic control and eventually inducing the onset of T2DM [2,76]. 

Uribarri et al. 2015 [77] conducted a cross-sectional study in a total of 269 obese subjects aged over 50 years with or without metabolic syndrome. Obese subjects with metabolic syndrome showed increased serum AGEs levels compared with obese subjects without metabolic syndrome. Homeostatic Model Assessment for Insulin Resistance (HOMA-IR), leptin, tumour necrosis factor α (TNF-α) and RAGE were associated with AGEs levels, while SIRT1, AGER1, glyoxalase-I and adiponectin were inversely associated with AGEs levels. The AGEs intake was higher in obese subjects with metabolic syndrome than in obese subjects without metabolic syndrome. These elevated AGEs levels were positively associated with dAGEs.

Acute studies [78,79,80] evaluated the effects of AGE meals on glycaemic control. Poulsen et al. 2014 [78] conducted an acute study of 19 healthy overweight subjects in randomised crossover design. They examined postprandial changes of satiety, inflammation and endothelial activation comparing a high-AGEs meal with a low-AGEs meal. The higher glycaemic response was observed after a high-AGEs meal than after a low-AGEs meal. A high-AGEs meal also elevated levels of urinary isoprostanes. No differences in visual analogue scale (VAS) scores for hunger, satiety, fullness and prospective food intake were observed between meals. No differences in blood levels of total ghrelin, glucagonlike peptide-1 (GLP-1) and peptide YY (PYY) were observed between meals. Blood levels of high-sensitivity C-reactive protein (hs-CRP), TNF-α, interleukin 6 (IL-6) and vascular cell adhesion molecule-1 (VCAM-1) did not differ between meals [78]. Schiekofer et al. 2006 [79] investigated acute effects of a casein meal with low- or high- AGEs contents on transcription factor nuclear factor-kappaB (NF-κB) in peripheral blood mononuclear cells (PBMC) of healthy subjects, which was independent of the AGEs content of the casein meal [79]. Negrean et al. 2007 [80] found higher postprandial levels of endothelial activation markers after a high-AGEs meal compared with a low-AGEs meal in subjects with T2DM. The higher flow-mediated dilation (FMD) for microvascular function was observed after a high-AGEs meal than after a low-AGEs meal even though both meals lowered FMD. Markers of endothelial dysfunction [plasma E-selectin, intracellular adhesion molecule 1 (ICAM-1) and VCAM-1] increased 2 h or/and 4 h after a high-AGEs meal compared with 2 h or/and 4 h after a low-AGEs meal. There were no significant differences in glucose, insulin, lipids [triglyceride (TG), total cholesterol (TC), low-density lipoprotein cholesterol (LDL-C) and high-density lipoprotein cholesterol (HDL-C)], inflammatory markers (hs-CRP, IL-6 and TNF-α) [80].

Meta-analyses of RCTs [81,82] examined the dAGEs on glycaemic control. A recent meta-analysis of 13 RCTs [10,11,12,13,15,16,17,19,83,84,85,86,87] conducted by Sohouli et al. 2020 [81] showed the favourable effect of a low-AGEs diet on metabolic syndrome risk factors such as insulin resistance, fasting insulin, TC and LDL-C. A low-AGEs diet showed significantly decreased concentrations of HOMA-IR [weighted mean difference (WMD) −1.204; 95% confidence interval (CI) −2.057, −0.358; *p* = 0.006] and fasting insulin (WMD −5.472 μU/mL; 95% CI −9.718, −1.234 μU/mL; *p* = 0.011), compared with a high-AGEs diet in quantitative analysis [81]. In lipid profile changes, a low-AGEs diet showed significantly decreased concentrations of TC (WMD −5.486 mg/dL; 95% CI: −10.222, −0.747 mg/dL; *p* = 0.023) and LDL (WMD −6.263 mg/dL; 95% CI −11.659, −0.866 mg/dL; *p* = 0.023), compared with a high-AGEs diet [81]. In a meta-analysis of 17 RCTs (22 publications [10,11,12,13,14,15,16,17,19,20,69,78,80,83,86,88,89,90,91,92,93,94]), Bay et al. 2017 [82] examined cardiometabolic risk by dAGEs in subjects with or without T2DM. Decreases in insulin resistance, TC and LDL-C were observed after a low-AGEs diet. Weight, fasting glucose, 2-h glucose and insulin, haemoglobin A1C, HDL-C or blood pressure (BP) were not changed after a low-AGEs diet. Especially, in subjects with T2DM, a low-AGEs diet led to reduced fasting insulin, TNF-α, VCAM-1, 8-isoprostane, leptin, circulating AGEs and receptor for AGEs. Adiponectin and sirtuin-1 were increased [82].

Several low-AGEs diet interventions of obese subjects showed improved insulin sensitivity [13,89,95]. Mark et al. 2014 [13] showed that a low-AGEs diet improved HOMA-IR after 4 weeks in 37 overweight women [13]. In a double-blind, randomised crossover trial of 20 healthy overweight subjects conducted by de Courten et al. 2016 [89], a low-AGEs diet improved insulin sensitivity as assessed by a hyper-insulinemic euglycaemic clamp and an intravenous glucose tolerance test, compared with a high-AGEs diet, even though no difference in body weight or insulin secretion was observed between diets. Two diets were isoenergetic- and macronutrient-matched diets with differences in AGEs contents [89]. Goudarzi et al. 2020 [95] examined the effects of a restricted AGEs diet on glucose metabolism, lipid profiles, oxidative stress (malondialdehyde) and inflammation (TNF-α and hs-CRP) in a RCT. Forty overweight subjects with metabolic syndrome completed either a calorie restriction and regular AGEs (R-AGEs) diet or a calorie restriction and a low-AGEs (L-AGEs) diet for 8 weeks. The L-AGEs diet significantly decreased CML, fasting glucose, fasting insulin, HOMA-IR, TNF-α and MDA compared with the R-AGEs diet. No significant difference in hs-CRP was seen between the two diets [95]. However, Linkens et al. 2022 [96] found no effect of a 4-week AGEs diet on glucose control and vascular function in 73 abdominally obese subjects (mean age 52 years; mean body mass index (BMI) 30.6 ± 4.0 kg/m^2^) with a double-blind parallel design of the intervention. A diet low in AGEs did not alter glucose, HbA1c, insulin, C-peptide, insulin sensitivity, secretion and clearance, micro- and macrovascular function (measured by FMD), inflammation, fatty liver index, estimated glomerular filtration rate (eGFR), lipid profiles (HDL-C, LDL-C and TG), oxidative stress and DNA glycation, compared with a diet high in AGEs. The gold standard ultra-performance LC–tandem mass spectrometry (UPLC-MS/MS) was used to assess AGEs and dicarbonyls in food, plasma and urine. Insulin sensitivity, secretion and clearance were assessed by a combined hyperinsulinemic-euglycaemic and hyperglycaemic clamp. No differences in urinary and plasma CML, CEL and Nδ-(5-hydro-5-methyl-4-imidazolon-2-yl)-ornithine (MG-H1) were observed between a low-AGEs diet group and a high-AGEs diet group [96].

A low-AGEs diet intervention of healthy subjects showed improved insulin sensitivity [97]. In an intervention of healthy subjects conducted by Birlouez-Aragon et al. 2010 [97], a high-heat-treated diet for 1 month significantly decreased insulin sensitivity, plasma levels of omega-3 fatty acids, vitamin C and vitamin E compared with a steamed diet. Moreover, reductions in TC, TG and HDL-C were observed after a high-heat-treated diet compared with a steamed diet [97]. A low-AGEs diet intervention of subjects with T2DM showed improved insulin sensitivity [17]. An AGEs-restricted diet for 4 months reduced HOMA-IR, insulin, leptin, TNF-α, NF-κB, p65 acetylation, serum AGEs and 8-isoprostanes in subjects with T2DM, compared with a standard diet [17].

In summary, given the recent intervention findings, a low-AGEs diet in healthy subjects, obese subjects and diabetic subjects appears to improve glucose homeostasis.

## 5. Dietary AGEs Influence on Body Weight

In epidemiological studies [Physical Activity, Nutrition, Alcohol, Cessation of smoking, eating out of home in relation to Anthropometry (PANACEA) study and a sub-cohort of the European Prospective Investigation into Cancer and Nutrition (EPIC) study] with 255,170 European subjects aged 25–70 years, the association between higher dietary AGEs intake and marginal greater weight gain was observed during the 5-year follow-up period. Especially, a high-CEL diet showed a 10% greater body weight increase compared with a low-CEL diet [98].

In a meta-analysis of 13 RCTs [10,11,13,16,17,19,83,84,85,86,87,89,99] conducted by the same research team of Sohouli et al. 2020 [100], a low-AGEs diet showed reductions in BMI (WMD −0.3 kg/m^2^; 95% CI −0.52, −0.09; *p* = 0.005; *I*^2^ = 55.8%), weight (WMD −0.83 kg; 95% CI: −1.55; −0.10; *p* = 0.026; *I*^2^ = 67.0%) and leptin (WMD −19.85 ng/mL; 95% CI −29.88, −9.82; *p* < 0.001; *I*^2^ = 81.8%), compared with a high-AGEs diet. Moreover, a low-AGEs diet showed significantly increased concentrations of adiponectin (WMD 5.50 µg/mL; 95% CI 1.33, 9.67; *p* = 0.010; *I*^2^ = 90.6%), compared with a high-AGEs diet.

Dicarbonyl stress is attributable from the abnormal accumulation of dicarbonyl compounds, which consequently promotes the development of cardiometabolic disorders including obesity by elevating protein and DNA modification [101,102,103,104]. Maessen et al. 2016 [105] examined whether a very low-calorie diet (VLCD) or Roux-en-Y gastric bypass (RYGB) decreased α-dicarbonyl stress in lean (n = 12) or obese women without (n = 27) and with T2DM (n = 27). Four groups [obese glucose-tolerant (NGT)-gastric banding (GB), NGT-RYGB, T2DM-RYGB and T2DM-VLCD] underwent mixed meal tests at baseline and 3 weeks at the end of intervention. Fasting and postprandial plasma α-dicarbonyl levels were higher in obese females with T2DM than in obese females without T2DM. Obese women with T2DM had significantly decreased postprandial α-dicarbonyl and fasting plasma α-dicarbonyls concentration after a 3-week VLCD [105]. Van den 2021 [106] conducted a weight loss intervention. Postprandial dicarbonyls [iAUC of MGO, GO and 3-deoxyglucosone (3-DG)] were decreased after an 8-week weight loss diet as compared with a habitual diet in abdominally obese men.

In summary, given the recent intervention findings, a low-AGEs diet appears to benefit body weight management.

## 6. Dietary AGEs Influence on Oxidative Stress and Inflammation

AGEs with oxidant properties elevate oxidative stress and inflammatory responses by stimulating RAGE in the AGE formation process, leading to the risk of cardiometabolic disorders [107,108,109,110,111].

Overweight and obese subjects (BMI 26–39 kg/m^2^) were randomly allocated to either a low-AGEs diet or a high-AGEs diet for 2 weeks in the intervention with a randomised crossover design. Two diets (9 MJ/day a diet) were isocaloric and macronutrient matched diets. A low-AGEs (3302 kU CML) diet improved inflammatory profiles [monocyte chemoattractant protein-1 (MCP-1) and macrophage migration inhibitory factor (MIF)] and renal function, compared with a high-AGEs (14,090 kU CML) diet [11]. Vlassara et al. 2016 [83] conducted a RCT of 100 obese subjects with the metabolic syndrome comparing a low-AGEs diet with a regular-AGEs diet for 1 year. After a low-AGEs diet compared with a regular-AGEs diet, HOMA-IR, fasting plasma insulin and insulin levels at 120 min of an oral glucose tolerance test (OGTT) were decreased. Body weight was slightly reduced. AGEs, oxidative stress (8-isoprostanes) and inflammation (TNF-α and VCAM-1) and RAGE mRNA decreased. The protective factors including mRNA levels of sirtuin 1, glyoxalase I and AGER1 increased. No differences in fasting blood glucose, HbA1c or blood glucose at 60 and 120 min of the OGTT were observed between two diets. Di Pino et al. 2016 [84] observed significantly decreased lipid levels of TC, apolipoprotein B (Apo B) and LDL-C after a 24-week low-AGEs diet compared with a standard-AGEs diet in 62 pre-diabetic subjects. Reductions in hs-CRP and intima-media thickness were observed after a low-AGEs diet, compared with the baseline of a low-AGEs diet. No change in arterial stiffness was observed in a within-diet group and a between-diet group. Semba et al. 2014 [15] performed a randomised, parallel-arm, controlled trial of 24 healthy subjects aged 50–69 years for 6 weeks. No differences in peripheral arterial tonometry, serum and urine CML, inflammatory mediators (IL-6, hs-CRP, VCAM1 and TNF-α receptors I and II), sRAGE and endogenous secretory receptors for AGEs were observed.

CML, one of primary dAGEs, stable and chemically inert AGEs, are unable to directly interact with tissue protein [112,113]. CML from a diet can be deposited in all tissues but adipose tissues. Kidneys, ileum, colon and lungs are organs with a higher deposition of CML, whereas the heart, liver and muscle are organs with a lower deposition of CML [114].

In vitro and in vivo studies showed the association-elevated inflammation and RAGE and CML-RAGE axis in obesity, consequently elevating insulin resistance [115,116]. In the Cohort on Diabetes and Atherosclerosis Maastricht Study and Hoorn Study performed by Gaens et al. 2015 [117], subjects with central obesity showed lower plasma levels of RAGE-mediated CML compared with lean subjects. The inverse association between RAGE-mediated CML and low-grade inflammation (LGI) scores was observed. These findings indicated in part “AGEs trapping (or AGEs build up)” in adipose tissue resulting in decreased levels of circulating plasma AGEs [117]. Ruix et al. 2021 [118] demonstrated that AGE/RAGE/diaphanous 1 (DIAPH1) axis in the immunometabolic pathophysiology of obesity was associated with insulin resistance partly throughout the expression and activity of this axis in abdominal subcutaneous (SAT) adipose tissue in obese subjects without T2DM [118]. CML does not always bind to RAGE for inflammatory response leading to cardiometabolic disorders, which indicates that dAGEs can promote inflammatory signalling cascade via a RAGE-independent pathway [112]. 

In summary, dietary AGEs are involved in increased oxidative stress and inflammation responses, which is associated with vascular dysfunction. 

## 7. Dietary AGEs Influence on Vascular Function

AGEs deposited in tissues and urine have been observed in cardiometabolic disorders and aging [119,120,121]. Two mechanisms of how AGEs contribute to tissue damage of cardiac or vascular dysfunction have been postulated: (a) AGEs can promote cardiac or vascular dysfunction by inducing inflammation and oxidative stress through receptors (Receptor-dependent way) [119]; (b) AGEs can elevate vascular and myocardial stiffness by cross-linking of elastin and collagen, consequently leading to vascular stiffness and cardiac fibrosis (Receptor-independent way) [122].

The RAGE, which is the most known human AGE receptor, is a multi-ligand type I cell surface receptor of the immunoglobulin (Ig) superfamily composed of three extracellular immunoglobulin domains, C1, C2 and V. Other AGEs receptors include scavenger receptors (e.g., stabilin-1, stabilin-2, SR-AI, SR-BI, OxLDL receptor 1, FEEL-1 and FEEL-2) and AGE-Rs (e.g., AGE-R1, AGE -R2 and AGE-R3) [123,124]. The expression of RAGE was found on different cells (endothelial cell, smooth muscle cells, chondrocytes, dendritic cell, fibroblasts, monocytes, microphages, T-lymphocytes, neuronal cell, glia cells and keratinocytes) [125,126,127,128,129,130]. Binding of AGEs to RAGE induces different intracellular signalling pathways, leading to elevated oxidative stress by increased intracellular ROS production with activation of NADPH oxidase, and activation of p21(ras)-dependent mitogen-activated protein kinase (MAPK) pathways triggering upregulation of NF-κB, its target genes and inflammation. The elevated levels of circulating cytokines (IL-1, IL-6 and TNF-α) contribute to a persistent inflammatory state [40,107,131,132,133]. 

Obesity was associated with decreased plasma levels of sRAGE leading to atherosclerosis risk [117,134,135,136,137,138,139,140,141,142]. The sAGE comprised of the ligand-binding site acts as a decoy receptor that inhibits the attachment of AGEs to RAGE [143]. Inhibition of AGE-RAGE binding can attenuate the development of atherosclerosis [143,144,145,146,147].

Rodríguez-Morteraa et al. 2019 [148] showed the effect of sRAGE on vascular function in a cross-sectional study of adolescents aged 15–19 years (33 obesity and 33 normal weight). sRAGE was associated with low-FMD and arterial stiffness index (Iβ). CML and AGEs were associated with atherogenic index (AI) [148].

AGEs accumulation sourced from food is associated with chronic inflammation, leading to risk of cardiometabolic disorders (especially, diabetic complication: risk of atherosclerosis in subjects with T2DM over about 10 years) [149]. The reactive dicarbonyl compounds, including glyoxal, MG and 3-deoxyglucosone, which are precursors of the primary quantitative AGEs, can directly influence extracellular matrix (ECM) modification [43,150]. The dAGEs can modify ECM. AGEs can be produced on proteins (collagen, laminin, elastin and vitronectin), and lipids in the ECM, which cause modification of ECM and stimulate increased stiffness [51,151,152,153]. The AGEs build-up on proteins in the ECM produces cross-links that can entrap other molecules [154]. AGE cross-linking on type I collagen and elastin can expand the area of ECM, leading to elevated vascular stiffness [51,151]. Moreover, AGEs build-up on proteins in the ECM can promote lipid-linked AGEs formation in subjects with or without T2DM [152]. Glycation of LDL-C in subjects with T2DM can decrease endothelial nitric oxide (NO) production and LDL-C clearance [153].

In the population-based cohort Maastricht Study of 2792 participants aged 60 years (26% T2DM) by Cordova et al. 2020 [155], the association between higher habitual intake of dicarbonyls methylglyoxal (MGO) and lower grade inflammation was observed after full adjustment, which was inversely associated with hs-CRP and TNF-α. Moreover, the association between higher dietary MGO intake and impaired retinal venular dilation was observed after full adjustment [155]. Linkens et al. 2022 [156] investigated the association between dietary AGEs intake and generalised microvascular function in a cross-sectional study (Maastricht Study) of 3144 participants aged 60 years. They determined dAGEs intake by combining the consumption of food items within the FFQ with their UPLC-MS/MS dietary AGEs database [30,156]. Microvascular function was evaluated with the retina [flicker light-induced arteriolar dilation, flicker light-induced venular dilation, central retinal arteriolar equivalent (CRAE) and central retinal venular equivalent (CRVE), the plasma endothelial dysfunction biomarkers [soluble VCAM-1, soluble ICAM-1, soluble E-selectin and von Willebrand factor(vWf)], skin (heat-induced skin hyperaemic response) and urine (24-h albuminuria). They did not find overall association between intakes of CML, CEL and MG-H1 and generalised microvascular function even though a higher CEL intake was associated with flicker light-induced venular dilation (β percentage change over baseline: 0.14; 95% CI 0.02, 0.26) and a lower plasma biomarker z score (β −0.04 SD; 95% CI −0.08, −0.00 SD) [156].

Linkens et al. 2021 [157] found intake of the dAGEs (CML, CEL and MG-H1) was not significantly associated with arterial stiffness as assessed by carotid–femoral pulse wave velocity (cfPWV), carotid distensibility coefficient (DC), and carotid Young’s elastic modulus (YEM) in the Maastricht Study [157].

In summary, the development of cardiometabolic disorders could be lowered by attenuating AGEs levels and RAGE expression and enhancing levels of sRAGE. The AGEs load can be reduced with high-AGEs diet restriction, low-AGEs cooking methods (low-temperature, boiling and steaming) and stopping smoking.

## 8. Dietary AGEs Influence on Gut Microbiome

Most of the dAGEs ingested orally are absorbed in the small intestine via the gastric tract and transported to the vascular system, while their remains are exported to the urine. The extent of dAGEs absorption depends on their chemical structures, molecular weights and hydrophobicity (e.g., pyrraline and argpyrimidine). Highly hydrophobic AGEs easily pass through the basolateral membrane compared with hydrophilic AGEs (e.g., CML, CEL and MG-H1) [34]. Non-absorbed AGEs are transported into the lower gut, where some are digested by gut microbiota, and the remains are exported via the faeces [46].

Western-style diets high in red meat, animal fat and/or simple sugar and low in fibre appear to negatively modify gut microbiota composition [158,159,160]. Several RCTs showed the effect of AGEs on gut bacterial microbiota [90,99,161]. Twenty male adolescents aged mean 12 years underwent a brown diet high in MRP content and a white diet low in MRP content in a two-week crossover design. A brown-diet group showed a reduction of enterobacteria correlated with increased intakes of hydroxymethylfurfural (HMF) and CML. *Lactobacilli* numbers were negatively associated with dietary advanced MRP such as hydroxymethylfurfural and CML. *Bifidobacteria* counts were negatively associated with Amadori compounds intake [90].

In a randomised, open label trial of 20 peritoneal dialysis patients, an AGEs-low diet for 1 month decreased *Prevotella copri* (considered a good bacteria [162]) and *Bifidobacterium animalis* compared with an AGEs-high diet. Moreover, an AGEs-low diet for 1 month increased *Alistipes indistinctus* (pathogenic causing colitis and site-specific tumours [163]), *Clostridium hatewayi*, *Clostridium citroniae* and *Ruminococcus gauvreauii* compared with an AGEs-high diet [99]. Linkens et al. 2022 [161] conducted a RCT with a double-blind parallel design to examine effects of 4-week isocaloric and macronutrient-matched AGEs diet on the gut microbial composition in 70 abdominally obese subjects (mean age 52 years; mean BMI 30.6 ± 4.0 kg/m^2^). They found the limited effects of dAGEs on microbiota composition. No difference in the Shannon index for microbial richness and diversity was observed comparing a low-AGEs diet with a high-AGEs diet. No difference in the overall microbial composition was observed comparing a low-AGEs diet with a high-AGEs diet. However, a low-AGEs diet decreased only *Anaerostipes* spp. abundance (one of 15 most abundant taxa in healthy individuals) compared with a high-AGEs diet. Moreover, the association between 3-deoxyglucosone (3-DG) intake and an abundance of several genera was observed [161].

In summary, limited studies showed dietary AGEs could play a potential role in disrupting gut microbiome and immune system. More interventions are required to clarify underlying mechanisms of action addressing the role of dAGEs in gut dysbiosis.

## 9. Skin AGEs as a Predictor of Cardiometabolic Disorders

**Figure 1:**
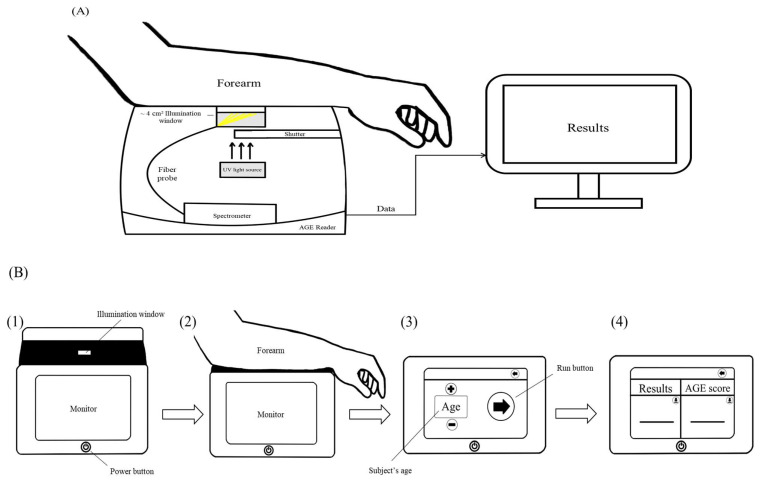
Description of the working principle and operating procedure of skin auto fluorescence (SAF) reader [184,185]. (**A**) Interior of a SAF reader is comprised of a scanner, an illumination window, a spectrometer, a result monitor and a fibre probe. (**B**) The working principle and operating procedure of advanced glycation end products (AGEs) measurement by SAF reader are as follows: (**1**) Measurement should be undertaken in a semi-dark environment. A reader is switched on by pressing power button; (**2**) A subject’s forearm should be placed on the scanner; (**3**) Subject’s information including age is entered into the reader, and then a run button is pressed. UV light from light source is illuminated with a maximum wavelength of 300–420 nm upon about 1 cm^2^ of skin, when the shutter is opened. This UV light activates autofluorescent AGEs in the skin of a subject’s forearm through the illumination window. The emitted light from the skin is transferred through the fibre probe. A spectrometer detects 420–600 nm fluorescence from light. The autofluorescence of the activated AGEs is assessed by the ratio between the emitted light intensity (420–600 nm) and the excitation light intensity (300–420 nm) multiplied by 100 on the integrated spectrometer; (**4**) AGEs scores (expressed in arbitrary units) from the spectrometer are reported as SAF value through the monitor.

The SAF is a non-invasive recent method to measure AGEs (Figure 1). The association between SAF and the quantity of certain AGEs in skin biopsies has been shown [65,66,67]. The levels of AGEs may markedly differ between tissue and plasma measurements. AGEs can be strongly deposited within the tissues (especially, dermal collagen), which can be measured through SAF considering certain AGEs have fluorescent properties. The SAF assessed by a Scout DS device showed clinically higher advantages than fasting glucose and HbA1c in subjects with undiagnosed abnormal glucose levels [164,165].

Meta-analysis conducted by Cavero-Redondo et al. 2018 [50] indicated that chronic accumulation SAF produced by enzymatic glycation in skin could be biomarkers of all-cause mortality and CVD mortality in subjects with diabetes and cardiovascular and/or renal diseases. This study showed the association between higher SAF and higher CVD mortality [hazard ratio (HR) 2.06; 95% CI 1.58, 2.67; *I*^2^ = 34.7%; *p* = 0.163] or higher all-cause mortality (HR 1.91; 95% CI 1.42, 2.56; *I*^2^ = 60.8%; *p* = 0.0.18) [50].

In a prospective cohort study with a 5-year follow up study period, the associations between higher SAF and elevated risk for all-cause mortality and elevated risk for fatal or nonfatal major adverse CVD events (MACE) were observed in subjects with peripheral artery disease [166].

Chen et al. 2022 [7] conducted a meta-analysis of 14 [1,73,166,167,168,169,170,171,172,173,174,175,176,177] prospective studies to examine if skin AGEs are associated with MACE. Skin AGEs were assessed by SAF. The significant associations between skin AGEs and elevated fatal CVD (HR 1.88; 95% CI 1.30, 2.70) and nonfatal CVD (HR 1.40; 95% CI 1.12, 1.74) were observed. In subjects with diabetes (HR 1.88; 95% CI 1.31, 2.69) and kidney disease (HR 1.50; 95% CI 1.16, 1.94), skin AGEs was associated with elevated MACE. This meta-analysis found the association between increased AGEs assessed by SAF and increased CVD events indicating skin AGEs could become a predictor of CVD events [7].

Several studies showed no association between skin AGEs and CVD events or death [73,168,169,170,171], while some studies showed the association skin AGEs and CVD events or death [177,178,179,180,181]. Waateringe et al. 2019 [177] conducted the Dutch Lifelines Cohort Prospective Study of 72,880 general non-diabetic population with a 4-year follow-up. They found the association between SAF and incident of T2DM, CVD and death independent of risk factors age, sex, waist circumference, metabolic syndrome, smoking and blood glucose levels. Especially, SAF was observed as the strong predictor for incident T2DM and/or CVD in subjects aged over 35 years.

Studies [182,183] showed higher SAF in subjects with metabolic syndrome compared to subjects without metabolic syndrome. SAF levels were independent of the presence of metabolic syndrome, waist circumference, impaired fasting glucose levels and BP.

## 10. Conclusions

A diet high in AGEs induces cardiometabolic disorders through increasing blood glucose and blood lipid concentrations, oxidative stress, inflammatory response, BP and body weight, compared with a diet low in AGEs. In addition, a diet high in AGEs may negatively change the gut microbiota and promote prevalence of cardiometabolic disorders compared with a diet low in AGEs. Further human studies using SAF assessments are needed to clarify the association between dAGEs in tissues and obesity, T2DM, CVD events, CVD mortality and total mortality.
